# Dynamic Evolution of Repetitive Elements and Chromatin States in *Apis mellifera* Subspecies

**DOI:** 10.3390/genes15010089

**Published:** 2024-01-11

**Authors:** Nick Panyushev, Max Selitskiy, Vasilina Melnichenko, Egor Lebedev, Larisa Okorokova, Leonid Adonin

**Affiliations:** 1 Institute of Environmental and Agricultural Biology (X-BIO), Tyumen State University, 625003 Tyumen, Russia; panyushev@nextmail.ru (N.P.); m.v.selickij@utmn.ru (M.S.);; 2 Bioinformatics Institute, 197342 St. Petersburg, Russia; larisa.okorokova@etu.univ-cotedazur.fr; 3 International Scientific and Research Institute of Bioengineering, ITMO University, 197101 St. Petersburg, Russia; vdmelnichenko@edu.hse.ru; 4 Institute of Biomedical Chemistry, Group of Mechanisms for Nanosystems Targeted Delivery, 119121 Moscow, Russia

**Keywords:** Hymenoptera, honeybee *Apis mellifera*, repetitive DNA, transposable elements (transposons; TE), noncoding RNA (ncRNA), chromatin landscape

## Abstract

In this study, we elucidate the contribution of repetitive DNA sequences to the establishment of social structures in honeybees (*Apis mellifera*). Despite recent advancements in understanding the molecular mechanisms underlying the formation of honeybee castes, primarily associated with Notch signaling, the comprehensive identification of specific genomic cis-regulatory sequences remains elusive. Our objective is to characterize the repetitive landscape within the genomes of two honeybee subspecies, namely *A. m. mellifera* and *A. m. ligustica*. An observed recent burst of repeats in *A. m. mellifera* highlights a notable distinction between the two subspecies. After that, we transitioned to identifying differentially expressed DNA elements that may function as cis-regulatory elements. Nevertheless, the expression of these sequences showed minimal disparity in the transcriptome during caste differentiation, a pivotal process in honeybee eusocial organization. Despite this, chromatin segmentation, facilitated by ATAC-seq, ChIP-seq, and RNA-seq data, revealed a distinct chromatin state associated with repeats. Lastly, an analysis of sequence divergence among elements indicates successive changes in repeat states, correlating with their respective time of origin. Collectively, these findings propose a potential role of repeats in acquiring novel regulatory functions.

## 1. Introduction

Honeybees play a vital role in preserving biodiversity and ensuring the food security of our planet [1]. They serve as essential pollinators for numerous crop species, facilitating their reproduction and yield [2]. Beyond their agricultural significance, honeybees also contribute to the maintenance of ecosystems and the balance of natural communities [3,4].

A honey bee colony consists of a queen, workers (females), and drones (males). The drones and the queen perform a reproductive function. The tasks of workers, associated with the upkeep of the colony, partly vary with their age [5]. Nurse workers build and clean the comb, store incoming pollen, protect the hive, and feed the newly hatched bees in their first weeks of life. Forager workers gather pollen, nectar, and water from their environment to provide for the colony. The caste development of social insects represents a significant evolutionary transition, playing a central role in their ecological success. Eusociality, which is defined as the division of labor, a shared care of offspring, and the presence of a sterile worker caste, has evolved repeatedly, especially among Hymenoptera. Each eusocial lineage is unique, with different social traits and its own evolutionary history [6,7]. A significant body of research data has accumulated on species with derived eusocial behavior, such as honeybees [8,9,10,11,12,13,14], but the genomic mechanisms regulating social behavior remain poorly understood.

Repetitive elements (REs), including transposable elements (TEs), are one of the main components of the eukaryotic genome. In insects, genome size and TE content can vary greatly [15]. Even among closely related *Drosophila* species, the TE content varies from 40% (in *Drosophila ananassae*) to 10% (in *Drosophila miranda* and *Drosophila simulans*) [16]. The highest TE content was found in the large genome of the migratory locust (*Locusta migratoria*, Orthoptera), exceeding 60% [17]. Previously seen as useless components of genomes, transposons, or “jumping genes”, are now recognized to have a significant influence on the evolution of the host genome’s structure [18,19]. Transposons have the ability to move and insert themselves into genes or regulatory sequences, disrupting coding sequences and gene regulation, resulting in chromosomal rearrangements [20]. Despite the potential negative impact on gene regulation, evidence suggests that TEs can also drive genomic innovations that offer advantages to the host [21,22,23,24,25].

REs are major constituents of long noncoding RNAs (lncRNAs, over 200 nucleotides) [26]. lncRNAs are involved in the epigenetic regulation of gene expression, acting as a regulatory link between chromatin-modifying complexes and the DNA sequence [27,28,29], maintaining loop interactions between promoters and enhancers [30], thereby acting as organizers of the three-dimensional architecture of the genome [31,32]. LncRNAs have been described to be involved in biological processes associated with honey bee behavior, such as dance behavior [33], sensory perceptions of odor, and olfactory receptor activity [34]. The rate of evolution of noncoding sequences has also been shown to correlate with major social transitions in bees [35].

Here, we relied on genomic and transcriptomic data available in public databases to assess the transcriptional activity of repetitive elements in the bee brain. We also analyzed the “historical” origin of repetitive elements and their domestication processes to assess the influence of repetitive elements on the regulation of eusocial behavior in the honey bee. 

## 2. Materials and Methods

### 2.1. RE Identification

The RepeatModeler program (v2.0.3) was utilized to identify de novo repeat elements in the reference honey bee genome for *A. m. mellifera*, GCF_003254395.2_Amel_HAv3.1 (Amel_HAv3.1), and for *A. m. ligustica* (GCA_019321825.1). Following that, the RepeatClassifier script and the Dfam3.6 database were employed to classify the consensus sequences. Subsequently, the obtained library of classified repetitive element (RE) families was used to mask the genomes of *A. m. mellifera* and *A. m. ligustica* using RepeatMasker with the -lib and -a options. To generate tab files with the percentage of divergence and Kimura plots, the resulting alignment output file was parsed through the parseRM script, https://github.com/4ureliek/Parsing-RepeatMasker-Outputs (accessed on 1 May 2023) (Figures S1–S3).

### 2.2. RNA-seq Analysis

Raw bulk RNA-seq reads ranging from SRR6727829 to SRR6727860 [36] were obtained from the SRA database and assessed for quality using FastQC (version 0.12.0). A pseudoalignment of the reads was performed using kallisto [37], utilizing an index generated from the reference transcriptome and consensus sequences of REs derived from RepeatModeler. A subsequent analysis was carried out in Rstudio using the DESeq2 (version 1.42.0) [38] R package. 

For single-cell RNA-seq analysis, the raw reads were downloaded from the SRA database (SRP338028) [39] using the fasterq-dump tool from the SRA-toolkit, pseudo-aligned using the Kallisto bustools (kb counts), and processed using the Seurat R package [40].

### 2.3. Genome Segmentation with ChromHMM

Datasets were downloaded from SRA [41]. To perform an initial quality control, we utilized FastQC and MultiQC (version 1.15). The reads from Chip-seq and ATAC-seq experiments were aligned to the HAv3.1 reference genome using STAR(version 2.7.11a) [42]. Afterwards, we employed ChromHMM [43] to binarize the aligned reads from Chip-seq (H3K27ac, H3K4me3, H3K4me1, and H3K27me3 histone modifications), ATAC-seq, and RNA-seq, with a 50-nucleotide genomic resolution.

Following the alignment and binarization steps, the combined data were utilized to train the model using the ChromHMM (version 1.25) LearnModel command. The input for training included 64 states (26 binary combinations), a bin size of 50, a file specifying the size of chromosomes, a file containing anchors and coordinates, and the model error change threshold.

Once the model was trained, the ChromHMM StatePruning command was executed to prune the model, resulting in 61 model files ranging from 2 to 63 states. The models were then compared to the original model using the ChromHMM CompareModels command. This comparison generated a graph that depicted the median and average loss values for each model in relation to the original model. Through an analysis of the obtained information, a model containing 23 states was chosen, as it covered 95% of the median losses observed in comparison to the base model.

Using the Python script with the pandas, numpy, and matplotlib libraries, a plot of emission and state transition probabilities was generated for the model, consisting of 23 states. Segmentation files for the three honey bee castes (worker, queen, and drone) were created using the ChromHMM MakeSegmentation command. From these segmentation files, a graph of the state transition probabilities, excluding transitions to itself, was constructed. Furthermore, based on this model, additional graphs were created. These included graphs depicting the starting site of transcription and the final site of transcription for each caste, as well as a graph displaying additional information for each state.

Based on the RNA-seq data, the coordinates of expressed and repressed exons and genes were determined. Additionally, the coordinates of known and unknown repeat classes were identified. The state in which these objects were located was determined using the ChromHMM OverlapEnrichment command (https://github.com/vasimel/Repeats_in_honeybee_genome/tree/apis_mellifera_model (accessed on 10 November 2023)).

To find information about genes intersecting with state 13 and the REs closest to them, the samtools intersect and samtools closest programs were used, respectively. To display graphs, a python script and the ShinyGO 0.77 program (http://bioinformatics.sdstate.edu/go/ (accessed on 1 December 2023)) were used.

## 3. Results

### 3.1. Expansion of Previously Uncharacterized Repeat Families Discriminate the A. m. mellifera and A. m. ligustica Subspecies

**Figure 1:**
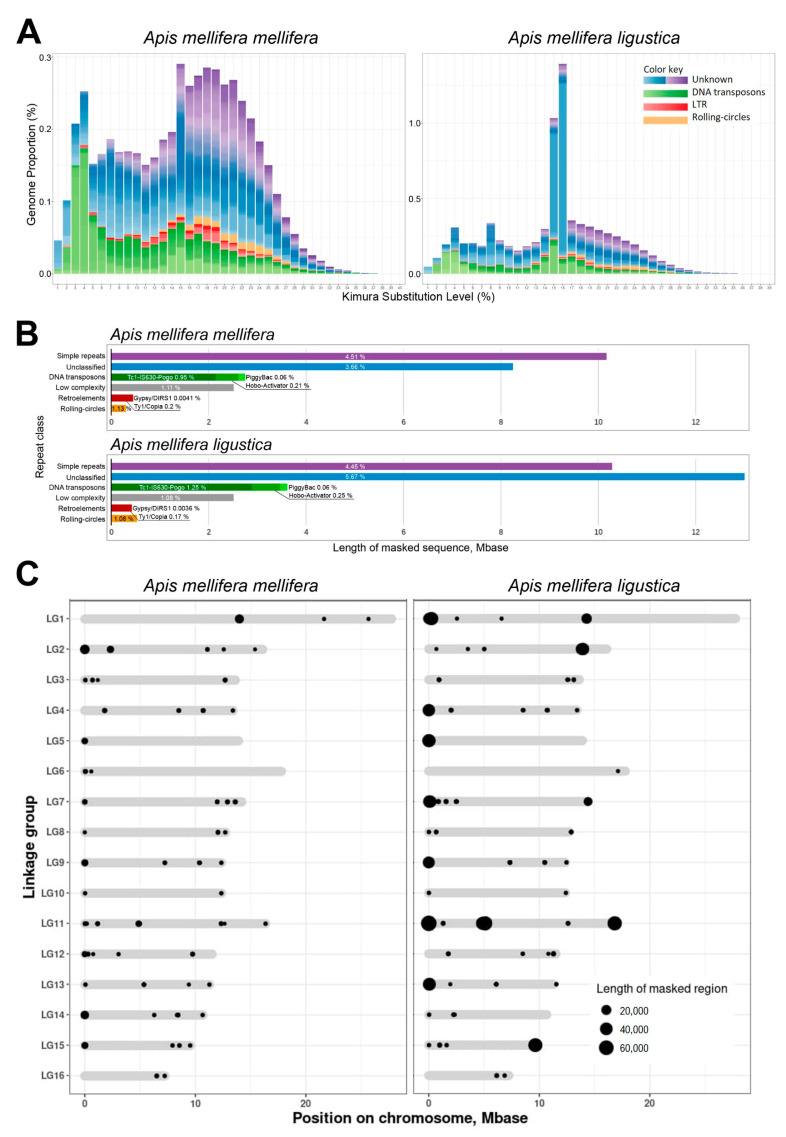
Pattern of repetitive elements in the *A. m. mellifera* and *A. mellifera ligustica* genomes. (**A**) Interspersed repeat landscapes. TE classes are marked with colors: DNA transposons, green; LTR TE, red; rolling-circle TEs (helitrons), yellow; unknown repeats, blue and purple; (**B**) the classification of repetitive sequences in the genomes; and (**C**) the distribution of rnd-4_family-321 elements on chromosome maps.

The Kimura plots presented in Figure 1A illustrate the evolutionary landscape of repetitive sequences in the genomes of the western honey bee, *A. m. mellifera* (*A.m.m.*), and one of its subspecies, *A. mellifera ligustica* (*A.m.l.*). 

The TE distribution pattern on the *A.m.m.* Kimura plot is characterized by two peaks located at 3–4% and 15–22% of substitutions from the transposable element (TE) consensus. The first peak is characterized by a high proportion of DNA transposon sequences, the highest in the whole diagram. The peak was preceded by a systematic increase in their copies in the previous stages. It is worth noting that, at this stage, which is relatively recent in evolutionary history, unclassified sequences contribute the least to TE diversity.

The second peak at 15–22% includes a great diversity of TEs, but the greatest contribution to this diversity is made by uncharacterized repeats. At this stage, there is an increase in DNA transposon diversity and the highest percentage of LTR elements, as well as the highest RC-Helitron copy number. The last two classes have almost no copies that have appeared in recent evolutionary history (1–2%).

The Kimura plot of *A.m.l.* has three peaks. The first peak at 4% includes copies of DNA transposons and unclassified repeats. The latter occupy less than half of the diversity at this stage. The second peak at 8% is characterized by a burst of diversity of uncharacterized sequences. The third peak at 15–16% is a distinctive feature of this genome compared to the *A.m.m.* genome, as it has the greatest amount of RE copies, but unfortunately, they cannot be characterized at the present stage of research. It can be seen that one single element has expanded the most at this peak—it is rnd-4_family-321. As in the *A.m.m.* genome, there are two peaks in the increase in DNA transposon diversity, corresponding to substitution levels of 3–4% and 15%. The absence of new copies of RC and LTR is shown, and their maximum diversity is noted at the level of 17–18%.

Repetitive elements (repeatome) account for 10.83% and 13.15% of the *A.m.m.* and *A.m.l.* genome sequences, as shown in Figure 1B. Simple repeats, low complexity, and unclassified repeats occupy the highest proportion of the genome. TEs represent 2.37% of the *A.m.m.* genome sequence, while for *A.m.l.*, TEs account for 2.8%. Retroelements are poorly represented in the compared genomes, and their percentage does not exceed 0.2% of *A.m.m.* and 0.17% of the *A.m.l.* genome sequence. Representatives of class II are the most abundant among all TEs. The percentage of the Tc1-IS630-Pogo superfamily in the *A.m.l.* genome is 1.25%, which significantly exceeds this index in the other subspecies (0.95%—*A.m.m.*). At the same time, the percentage of another group of DNA transposons, helitrons, is comparable in the studied genomes, and equals 1.13% and 1.08% in *A.m.m.* and *A.m.l.*, respectively.

Figure 1C shows the expansion of rnd-4_family-321 in the centromere and telomere proximal regions of the chromosomes of both subspecies. This specific repetitive element is scientifically intriguing due to its considerable expansion in the genome of *A.m.l.* It is noteworthy that it comprises several copies of AvaI and AluI repeats, making up a longer and more intricate structure.

In both genomes, rnd-4_family-321 elements are mostly located in regions proximal to telomeres and centromeres. These elements grew in size mostly in the telomeres of *A.m.l.* chromosomes (LG1, LG4, LG5, LG7, LG9, LG11, LG13, LG15).

### 3.2. Integrating Multi-Omics Data via Markov Models for Genome-Wide Chromatin State Assignment

**Figure 2:**
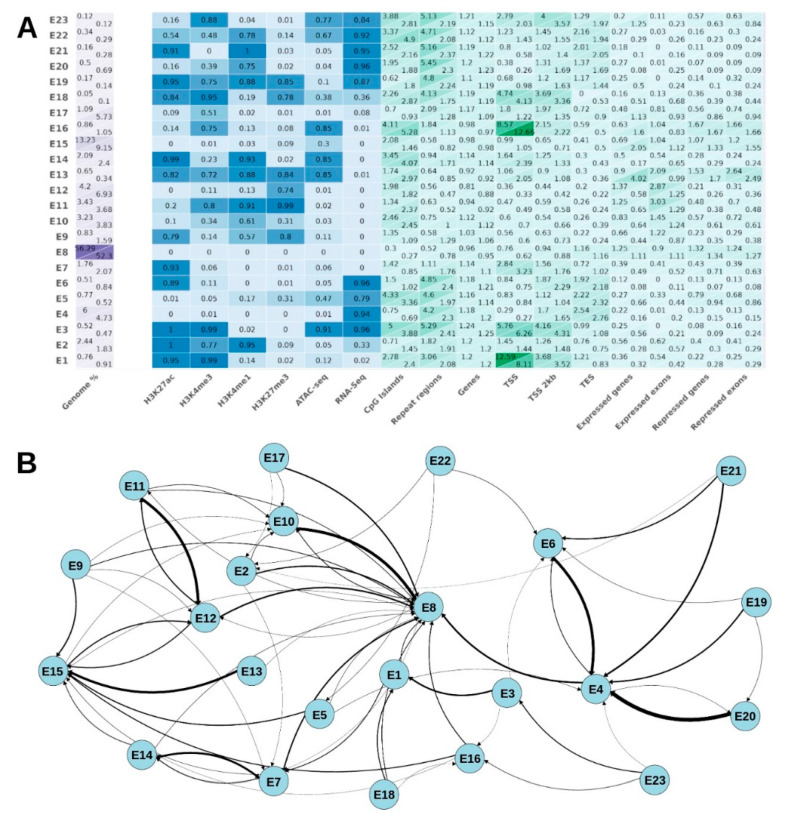
Features of chromatin states and genome occupancy in *A. m. mellifera* brains. (**A**) Features of the different chromatin states in queens/drones. Columns from left to right—occupied genome fraction (purple), features comprising states (in blue), and relative enrichment of respective genomic regions (in green); (**B**) the state transition graph; transition probabilities < 0.05 are not shown. The edge thickness indicates the probability of transition.

In our study, we employed Chip-seq data for four distinct histone marks (H3K27ac, H3K4me3, H3K4me1, and H3K27me3) along with RNA-seq and ATAC-seq data. The purpose of using these datasets was to train a ChromHMM model and subsequently refine it through a process called model pruning. By applying this approach, we successfully identified a total of 23 distinct chromatin states (Table S1). The features enriched in each chromatin state are similar for all castes (Figure 2A for queens and drones and Figure S5 for workers).

The distinct enrichment of H3K4me3 and H3K27ac, along with depletion of H3K4me1 and H3K27me3, is clearly evident in E1 and E3 (E before the state number is our internal designation) chromatin states, allowing them to be grouped together as TSS-associated states [41]. E1 and E3 are likely to represent two alternative states of TSS—inactive and active ones, respectively.

E2 and E22 also show enrichment in H3K27ac and H3K4me1, as well as a slight enrichment in the H3K4me3 label, which may be characteristic of intron regions [44]. According to the state transition graph (Figure 2B), E22 can transit to the E2 state along with losing RNA-seq features. Taking this into account, we can suggest an active exon feature for E22 and E2 as the intronic regions of those genes. E20 has a very similar pattern of features to E22, but shows no transitions to neither E22 nor E2, likely to be highly expressed housekeeping genes. The E19 state is enriched in all inherent histone marks, but has a low level of ATAC-seq. E19 shows a high association with expressed exons. E19 can only transit to the E6 or E20 state, making it also closely associated with gene expression. Potentially, the E19 state represents poised promoters, containing marks characteristic of both active and inactive chromatin. Both E21 and E14 are marked by high levels of H3K27ac and H3K4me1, while being depleted in H3K4me3. The H3K4me1 mark correlates with enhancers [45], while H3K27ac indicates their active state [46]. E21 also shows a high enrichment in RNA-seq, and is also associated with exons. On the contrary, the E14 state shows no association with expression and exon regions, but it is associated with chromatin accessibility. Thus, in our opinion, E21 and E14 could be seen as enhancers. E9, in addition, shows a high level of H3K27me3, which characterizes this condition as a poised enhancer [45].

The E4 state and, to a lesser extent, the E5 state are characterized by the almost complete absence of histone marks, but by the enrichment of RNA-seq. Due to the transition graph, the E4 and E5 states can only transit to inactive states (E8, E15). Thus, they can be treated as states with residual background expression. 

The E6 and E7 states are characterized by H3K27ac enrichment and low levels of H3K4me3, H3K4me1, and H3K27me3. E6 shows a strong association with transcribed genes. E7 shows no association with genes, but the E14 state can transit to E7 and revert back, hinting that it can be a potential cis-regulatory element in the switched-off state. 

E8 shows a strong depletion in all features used and is present in the highest genome fraction (56.3%), being labeled as an extensive silent domain [44]. The E15 state of chromatin shows a similar label distribution, except for a slight enrichment in ATAC-seq.

The E10 state has a moderate enrichment in H3K4me1, with weak H3K4me3 and H3K27me3. Based on the transition graph, it can change only to E8, along with losing all marks. Based on this evidence, we can characterize it as the transition state. On the contrary, E11 has the strongest enrichment in H3K27me3, suggesting its inactivation, along with strong enrichments in H3K4me1 and H3K4me3. It also shows a strong affinity to repressed exon features. This indicates that these are regulatory elements of repressed genes.

The E10 and E11 states show a similar enrichment of the H3K4me3, H3K4me1, and H3K27me3 labels, and a low level for the rest of the labels. They do not show any association with exons/genes. They are likely to be transitory states of sorts. 

The E12 state is distinguished by a high enrichment of the label with H3K27me3, which is characteristic of regions of Polycomb-mediated repression [44].

E16 has a strong association with TSS regions, and the strongest association with CpG islands. Also, this state is most tightly associated with repressed genes. Taken together, this state demarcates the repressed gene transcription start sites. 

The region referred to as E17 exhibits a moderate enrichment in the histone mark H3K4me3, which is associated with active gene transcription. Meanwhile, it displays a depletion or decrease in other types of histone modifications.

Finally, E18 is characterized by a low level of H3K4me1, but together with a high level of H3K27ac, H3K4me3, and H3K27me3. Together with low but not absent ATAC-seq and RNA-seq signals, this can suggest this state to be the switched-off promoter. This assumption is strengthened by the transition graph, because it can transit to the inactive TSS-associated states (E1 and E16). Therefore, together with E23, E18 states represent the smallest fraction of the genome in workers (0.09% and 0.08%, respectively). 

E23 is quite poorly represented, comprising no more than 0.09% of the total genome in the worker caste, representing the smallest fraction. This is likely to be the transition state for TSS regions. If they lose their RNA-seq signal, they will become repressed, similar to E16, or, alternatively, transit to the active condition, together with H3K27ac (like E3).

### 3.3. REs Are Significantly Enriched in Special Chromatin State (E13) 

**Figure 3:**
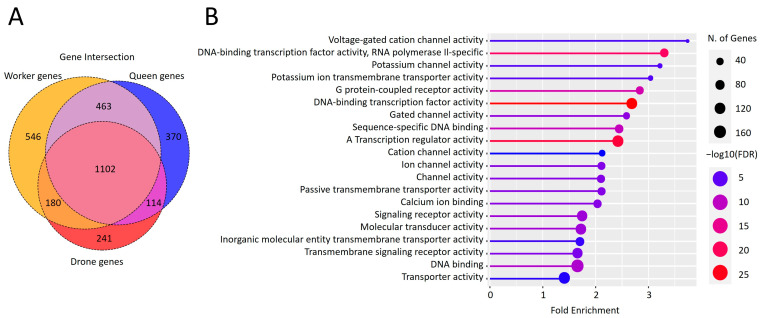
Localization features of E13 chromatin state regions and GO analysis of their closest genes. (**A**) Venn diagrams indicating intersection between closest genes to E13 regions in different castes. (**B**) Lollipop diagram indicating the Gene Ontology molecular function enrichment of closest genes to E13.

For E13 (Figure 3), state enrichment in all histone marks is inherent, but it has no transcription activity. This state is, however, the unique one, specifically enriched with repeats. This specific state can only transit to E15 and E8, believed to be non-active states.

### 3.4. REs Demonstrate Opposing Trends in E8 and E15 Chromatin State Proportions within 0–3 and 20–40 Kimura Distances

**Figure 4:**
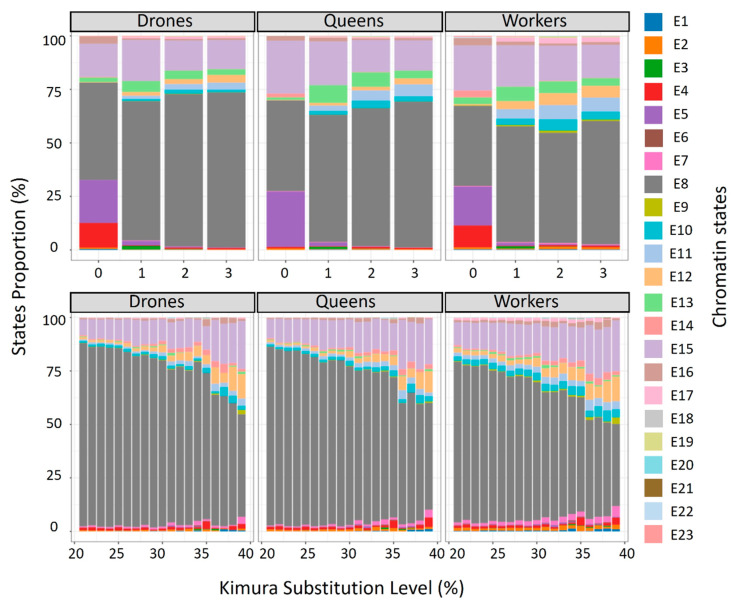
Bar plots depicting chromatin states in young (Kimura 0–3) and middle-aged (Kimura 20–40) repeats. (**Top**) Young repeats; (**bottom**) middle-aged repeats. Stacked bar graphs illustrate the proportional genome occupancy of each chromatin state across various Kimura distance intervals.

In the E8 state, characterized as having extensive silent domains, an increase in chromatin state proportions is observed as the Kimura substitution level increases (Figure 4 top). However, for higher Kimura levels (Figure 4 bottom and Figure S4), the trend changes towards a decrease in proportion, and at the same time, an increase in the E15 state—close to E8, but more enriched in ATAC-seq.

### 3.5. Transcriptional Activity of Repetitive Elements during the Larval Stage in Queens and Workers

The most actively expressed repeats are unclassified ones: rnd-1_family-83 (baseMean = 12.28) and rnd-6_family-3082 (baseMean = 11.93). Among the classified elements, DNA elements are the most actively expressed (rnd-5_family-1919#DNA/CMC-EnSpm (baseMean = 10)) as well as Mariner elements (rnd-1_family-1 (baseMean = 9.23), rnd-6_family-371 (baseMean = 7.69), and rnd-6_family-1748 (baseMean = 7.03)).

**Figure 5:**
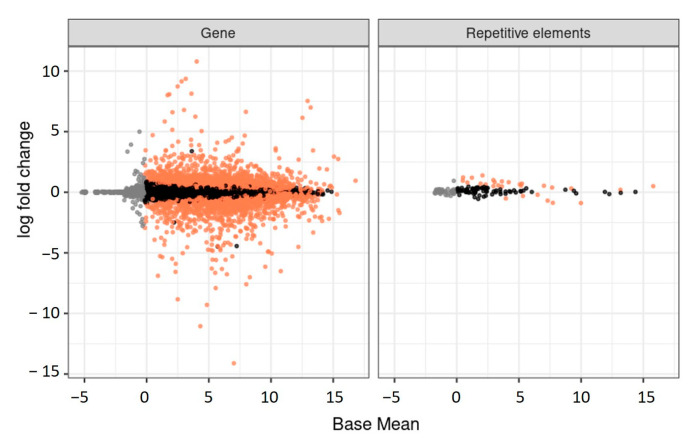
The MA plot visualizes expression differences of the genes and repeats in queen and worker larvae. The *x*-axis represents the average expression level, while the *y*-axis depicts the log-fold change, highlighted with orange—transcripts with differential expression.

Compared to some genes that are expressed differentially in queens and workers, repetitive elements have much less difference in expression (max. log fold change is 1.4 for an unknown repeat, rnd-1_family-6). Some DNA elements, however, are differentially expressed in the brains of larvae: rnd-5_family-1919#DNA/CMC-EnSpm is downregulated in queens (log2 fold change = 0.9, *p*-value < 0.05); rnd-1_family-78#DNA/TcMar-Mariner is upregulated, with log2 fold change = 0.71 (*p*-value < 0.05), as well as rnd-1_family-11#DNA/TcMar-Mariner, with log2 fold change = 0.67 (Figure 5).

### 3.6. Expression of Eight Repeats Segregates Brain Cell Populations

**Figure 6:**
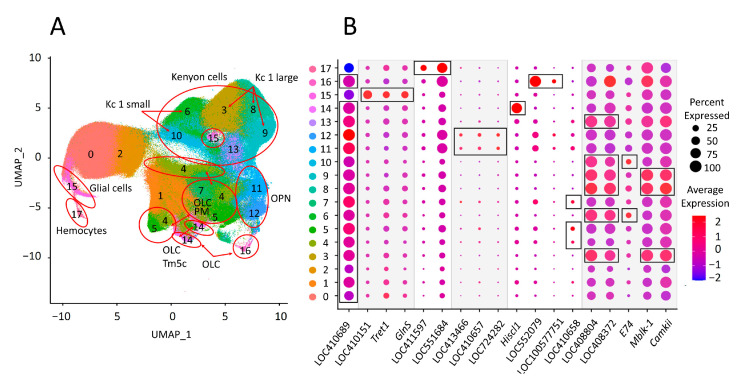
Clustering of single-cell transcriptome of the honey bee brain. (**A**) The UMAP of single cells from the brain of a honey bee, divided into 5 main clusters: hemocytes; glial cells; olfactory projection neurons (OPNs); optic lobe cells (OLCs); and Kenyon cells (KCs). OLCs were subdivided into Tm5c, Lawf2, and PM neurons. Kenyon cells were subdivided into class I small KCs and class I large KCs. (**B**) Dot plot of predictive gene markers used for cluster annotation. The black frame shows the marker/combination of markers used to identify the cell population.

A total of five main cell populations were identified (Figure 6A). To identify glia (cluster 15), the marker genes LOC410151 (*repo*) [47], *Tret1,* and *GlnS* [48] were used. Hemocytes (cluster 17) were identified using markers LOC411597 (*hml*) and LOC551684 (*fer2LCH*) [49]. Cells expressing the neuronal marker *elav*, the functions of which have been shown for *D. m.* [50] (LOC410689), were defined as neurons (all clusters except 15 and 17). These cells are divided into olfactory projection neurons (OPNs), optic lobe cells (OLCs), and Kenyon cells (KCs) (Figure 6B). 

OPNs: The axons of the olfactory receptors of the honey bee project into the lobes of the antennae, which consist of glomeruli. The glomeruli are connected to each other by local interneurons, and from the glomeruli, projection neurons are directed to the center of the brain of a higher order, such as the mushroom bodies and the lateral protocerebrum [51]. Using a combination of markers (LOC413466 (*oaz*), LOC410657 (*acj6*), and LOC724282 (*opt*)), we identified two clusters of olfactory projection neurons (clusters 11 and 12) [49,52].

OLCs: The optic lobes of the honey bee are responsible for the transduction of light stimuli and conductivity to mushroom bodies, where they evoke a reaction [53]. *Hiscl1* is highly expressed in cluster 14 and is associated with the type of Tm5c neurons. We also identified a population of lamina wide-field neurons of the second type—Lawf2 (cluster 16; LOC552079 (*hth*), LOC100577751 (*lim1*)). Finally, using the marker LOC410658 (*lim3*), we identified the PM cell type of the optic lobe (clusters 4, 5, and 7) [39,49].

KCs: The mushroom bodies are the processing centers for sensory information and are also involved in learning and memory [54]. We found five mushroom-body cell clusters (clusters 3, 6, 9, 10, and 13; LOC408804 (*plc*) and LOC408372 (*mub*)) [39].

Mushroom-body Kenyon cells are divided into two classes based on their morphology, function, and localization [52]. In addition, the first class is subdivided into three subclasses (small, medium, and large), and the population of FoxP-expressing cells is described separately [55]. Also, class I small KCs (clusters 6, 10; *e74*) and class I large KCs (clusters 3, 8, 9; mblk-1, cAmKii) [56] were identified. Class 1 medium cells expressing *mKast* gene and FoxP population cells were not found due to a lack of the necessary marker genes.

### 3.7. Repetitive Elements

**Figure 7:**
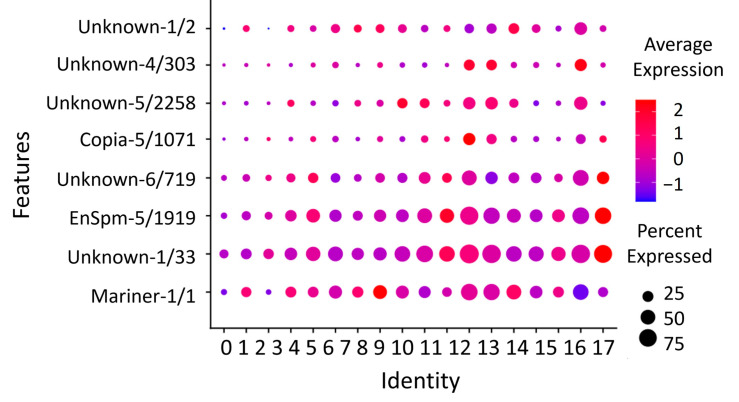
Dot plot of eight REs predicted as markers. Among the identified markers, the highest expression level (average expression 2.5) was observed for the Copia-5/1071 in cluster 11, which was identified as olfactory projection neurons. Additionally, the EnSpm-5/1919 showed high expression in cluster 17, characterized as hemocytes.

Eight REs have been identified as markers of cell populations (Figure 7; Table S2). Unknown-1/2 is expressed in clusters 6, 13 (KCs), and 7 (OLCs). Unknown-4/303 is expressed in 11, 12 (OPNs), and 16 (OLCs). Unknown-5/2258 is expressed in three, eight, and nine clusters, identified as class 1 large KCs. Copia-5/1071 is expressed in 11 (OPNs) and 17 (hemocytes). Unknown-6/719 is expressed in four (OLCs), 10 (KCs), and 17 (hemocytes). EnSpm-5/1919 and Unknown-1/33 show a similar expression pattern in 10 (KCs) and 17 (hemocytes). Hemocytes play a crucial role in the cell-mediated immunity of insects [57]. They are responsible for various functions, such as detecting infectious agents, the phagocytosis of small particles, and the encapsulation of larger foreign objects [58]. Mariner-1/1 is found in two clusters: 7 (OLCs) and 13 (KCs).

## 4. Discussion

### 4.1. Eusociality

Eusociality represents an intricate form of social conduct marked by a division of labor between reproductive and non-reproductive castes for achieving a highly structured and cooperative social organization in the natural world. This high level of social organization has independently emerged in numerous Arthropod taxa, including Decapoda (shrimps), Isoptera (termites), Aphididae (aphids), Thysanoptera (thrips), Coleoptera: Scolytidae (bark beetles), and Platypodidae (ambrosia beetles) [59,60,61,62,63], and particularly in Hymenoptera (sawflies, wasps, bees, and ants) [64,65,66,67]. The origin of eusociality is estimated to have occurred at least fifteen times within the aculeate Hymenoptera, without taking into account a sole origin amongst ants [7]. The development of eusociality represents a significant adaptive advantage for these species and has culminated in their global prevalence and diverse ecological roles today. The evolutionary origins of eusociality have attracted biologists’ interest for many years, resulting in several theories that explore various evolutionary processes. The precise emergence of eusociality in Hymenoptera cannot be pinpointed in the fossil record, rendering the timeline a subject of ongoing analysis. However, according to phylogenetic studies and molecular clock analyses, it is estimated that eusociality emerged approximately 100 million years ago during the Cretaceous period [68].

### 4.2. Distribution of TEs

Transposon-mediated gene regulation is likely one of the mechanisms underlying the eusocial transition [69]. Although eusocial species of shrimps [59], cockroaches, and termites [70] have been found to have a higher number of transposons, bees show a reduced quantity in conjunction with smaller genome sizes. The decrease in the number of transposable elements observed in eusocial bees may be a consequence of their social life, and possibly linked to a balance between genomic diversity and integrity, driven by recombination and TE suppression [71,72].

Repetitive elements comprise a significant proportion of the genome in many metazoans. While repeatomes are usually similar in closely related organisms, their content and landscape may significantly vary in different representatives of one genus [73]. Moreover, differences in repetitive element distribution can be observed between closely related species [16], which is caused by the expansion and elimination of repetitive element copies. The expansion of the AluI element observed in the genome of *A. m. ligustica* suggests that REs are constantly evolving and may be the source of intraspecies variations.

The honey bee contains an unusually low amount of repetitive DNA. Many elements are fragmentary and incomplete, and although they may contain sequences of typical transposable element protein domains, they are often rendered inactive by stop codons and frameshift mutations [74]. Social complexity in bees has been shown to negatively correlate with the abundance and diversity of transposable elements (TEs) [16,18].

In multicellular organisms, a number of protective mechanisms guard against the uncontrolled proliferation of transposable elements. These defense systems, particularly piwi-interacting RNA (piRNA) [75,76] and zinc-finger genes [77,78], are notable among these mechanisms. They adapt to changing transposable elements, thereby applying selection pressure on the TEs [79,80]. Major biological shifts frequently involve the expansion of transcription factor families [81].

Repetitive elements have the potential to evolve into cis-regulatory elements [82]. In mammalian genomes, transposons are increasingly recognized as a significant source of diverse cis-regulatory sequences. For instance, in mice and humans, LTRs linked to several pluripotent transcription factors are commonly found to be enriched with enhancer-associated chromatin signatures such as H3K27ac and H3K4me1, indicating their role in the regulation of gene expression [83].

### 4.3. Limitations of ChromHMM

Our study has several technical limitations. The alignment of the results from a biological experiment with sequencing reads of 150 bp does not provide 100% accuracy, as the repetitive elements in the genome of *A. mellifera* are lengthy, with an average length of 343.4 bp and a maximum length of 11,867 bp. Aligning genome pieces with 150 bp reads cannot cover the length of REs, leading to low alignment accuracy for features such as Chip-seq and RNA-seq regions with REs [84]. The genome resolution of 50 bp for the mean or median value also hinders achieving high accuracy. The limited number of available features (ATAC-seq, RNA-seq, and four modified histones) can obscure potentially active regions of the genome that may play a role in certain processes. For example, adding a new feature can decrease state 8 in our model and expand another state, or even introduce a new state, thereby describing a greater number of genome regions with non-silent states [43,85]. The above factors can significantly impact the final result of clustering and analysis. Improving alignment accuracy, increasing the length of sequenced reads to cover repetitive elements of the genome (for instance, by using Oxford Nanopore), incorporating more features (a broader range of histone modifications, for example, the histone group, h1, h2a, h2b, h3, h4, and their modifications such as h3k9me3, as well as other features like RNA-seq, atac-seq, and wgbs), and exploring other clustering methods can enhance the accuracy and resolution of the methods [85,86].

Overall, the combination of proposed improvements could increase the accuracy and resolution of the methods, even if they do not fully overcome the problem of repetitive elements and allow more genomes to be clustered with greater accuracy.

### 4.4. REs Markers in scRNA-seq Data

Eight TE sequences were identified as markers in different populations of scRNA-seq data from the adult honey bee brain. The limited number of markers can be attributed to the low activity of transposable elements in adult tissues [87] and the relatively low abundance of TEs in the bee genome compared to other insects [14]. Studies have shown patterns of transposon expression in the adult *Drosophila* brain [88], but there is still insufficient data to identify expression patterns of mobile elements and their potential role in caste determination in bees.

## 5. Conclusions

Based on our extensive investigations, we have concluded that the repetitive elements present in the *A. m.* species are associated with the divergence observed among its subspecies. Despite the recent expansion of these repetitive elements, our analysis suggests that they have no discernible effect on caste formation at the transcriptomic level. However, our examination of the chromatin landscape has revealed the existence of a distinct state, specifically associated with these repetitive elements. This discovery, combined with the apparent process of “domestication”, serves as the basis for uncovering compelling evidence for the direct involvement of genomic repeats in the evolution of social behavior. The emergence of a unique chromatin state associated with repetitive elements provides preliminary evidence for the potential involvement of repeat-derived sequences in the evolution of *A. mellifera*. The ongoing dynamics of domestication provide an avenue for this involvement in the immediate evolutionary future.

These findings shed new light on the complex mechanisms underlying the evolution of social behavior and provide valuable insights into the role of repetitive elements in shaping the genomic landscape of honey bees.

## Data Availability

Data are contained within the article and supplementary materials.

## Supplementary Materials

The following supporting information can be downloaded at https://www.mdpi.com/article/10.3390/genes15010089/s1: Figure S1: Scatter plot of repeat coverage vs repeat copy number in genome using different modes of alignment. Every dot depicts a distinct repeat element. The x-axis depicts the repeat copy number and the y-axis depicts the median coverage of these elements. Blue colour dots—the unique alignment mode, ignoring all the multi-mapper hits. Orange dots represent values obtained with the multialignment mode, reporting all the hits for every element; Figure S2: Boxplot of repeat coverage comparing the alignments in multimapper mode and single mapper mode. To the right—the unique alignment mode, ignoring all the multi-mapper hits. The left box represents values obtained with the multi alignment mode, reporting all the hits for every element; Figure S3: Density plot of the lengths distribution of the repeats of different classes identified by RepeatModeler; Figure S4: Bar plots of chromatin states in repeats throughout the total range of Kimura distances Stacked bar plots indicate the occupied fraction of the genome by each chromatin state for every Kimura distance window value; Figure S5: Features of chromatin states and genome occupancy in brain of the workers of A.m.mellifera. Columns from left to right—Occupied genome fraction (purple), Features comprising states (in blue), and relative enrichment of respective genomic regions (in green); Table S1: ChromHMM chromatin states interpretation; Table S2: Repeats, which serve as cluster markers in sc-RNA-seq analysis. Average expression shows the mean counts by all cells in a given cluster. Percent expressed shows the portion of cells expressing the repeat.
