# Supplementary material for: Dynamic Evolution of Repetitive Elements and Chromatin States in Apis mellifera Subspecies
Source: Genes (Basel). 2024 Jan 11;15(1):89. doi: 10.3390/genes15010089 (PMC10815273; doi:10.3390/genes15010089)
Supplement: Supplementary file 1 [file genes-15-00089-s001.zip › genes-2801171-supplementary.pdf]

Supplementary materials

Dynamic Evolution of Repetitive Elements and Chromatin States in *Apis mellifera* Subspecies.

Panyushev N., Selitskiy M., Melnichenko V., Lebedev E., Okorokova L. and Adonin L.

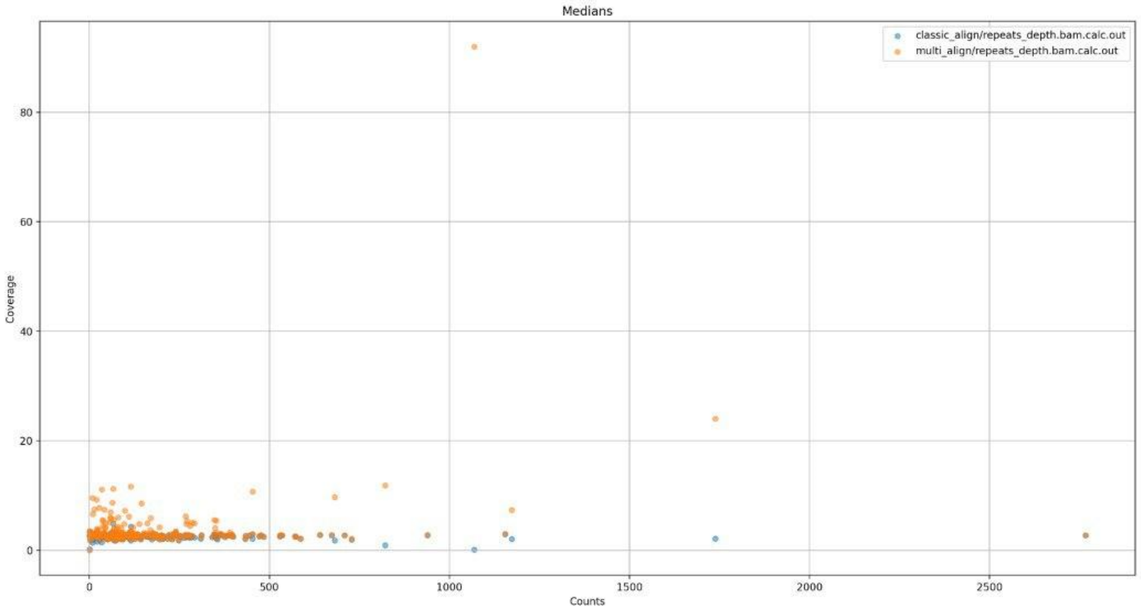

Figure S1. Scatter plot of repeat coverage vs repeat copy number in genome using different modes of alignment. Every dot depicts a distinct repeat element. The x-axis depicts the repeat copy number and the y-axis depicts the median coverage of these elements. Blue colour dots - the unique alignment mode, ignoring all the multi-mapper hits. Orange dots represent values obtained with the multialignment mode, reporting all the hits for every element.

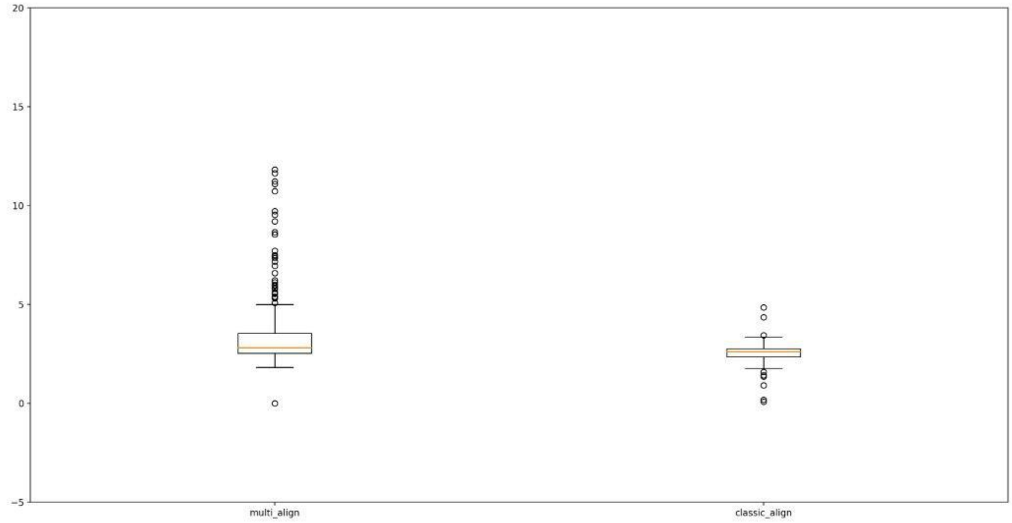

Figure S2. Boxplot of repeat coverage comparing the alignments in multimapper mode and single mapper mode. To the right - the unique alignment mode, ignoring all the multi-mapper hits. The left box represents values obtained with the multi alignment mode, reporting all the hits for every element.

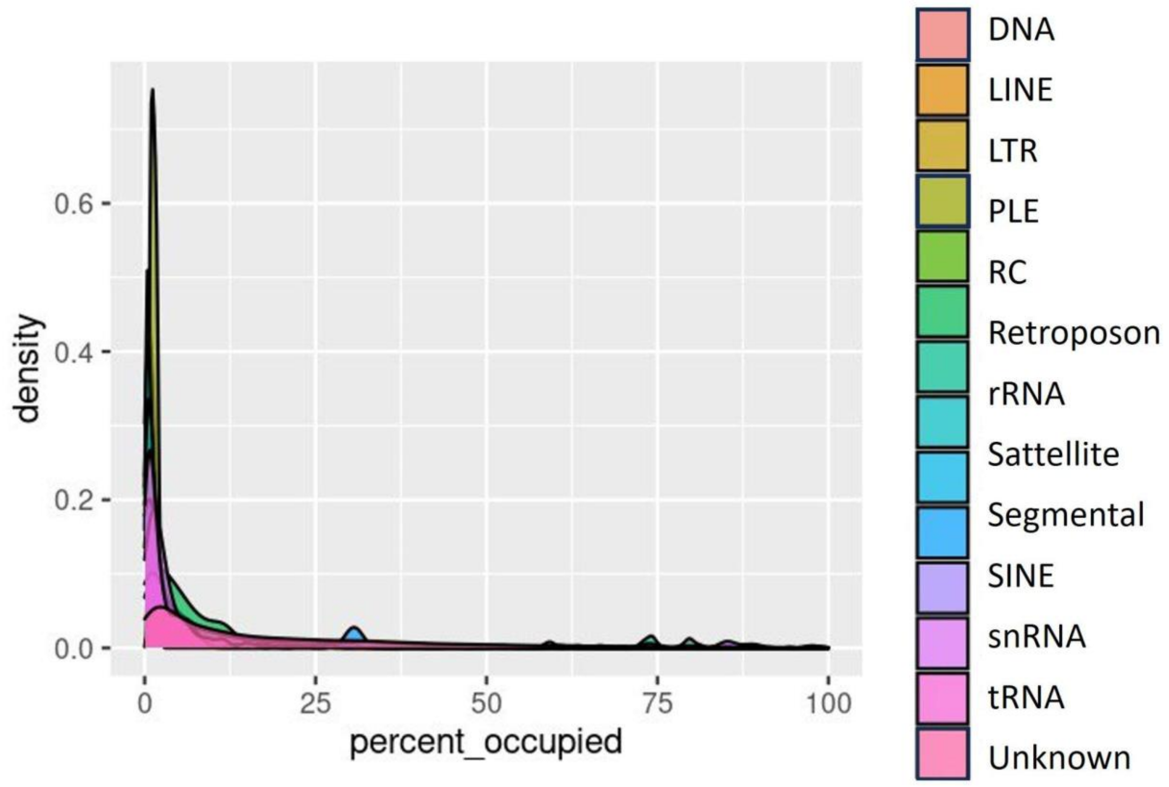

Figure S3. Density plot of the lengths distribution of the repeats of different classes identified by RepeatModeler

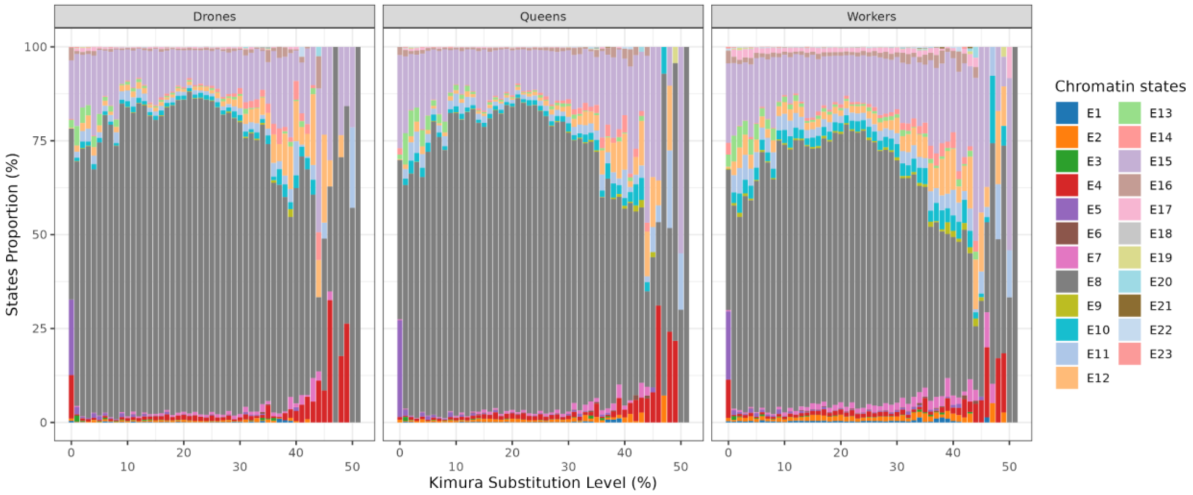

Figure S4. Bar plots of chromatin states in repeats throughout the total range of Kimura distances Stacked bar plots indicate the occupied fraction of the genome by each chromatin state for every Kimura distance window value.

	Genome %	H3K27ac	H3K4me3	H3K4me1	H3K27me3	ATAC-seq	RNA-Seq	CpG Islands	Repeat regions	Genes	TSS	TSS 2kb	TES	Expressed genes	Expressed exons	Repressed genes	Repressed exons
E23	0.09	0.16	0.88	0.04	0.01	0.77	0.84	3.72	0.53	1.18	2.05	3.65	2.19	3.4	5.63	0.33	0.16
E22	0.36	0.54	0.48	0.78	0.14	0.67	0.92	2.92	0.19	1.14	1.11	1.51	2.14	3.25	5.36	0.33	0.02
E21	0.13	0.91	0	1	0.03	0.05	0.95	2.13	0.07	1.19	0.65	1.2	2	3.39	5.89	0.34	0
E20	0.34	0.16	0.39	0.75	0.02	0.04	0.96	1.6	0.09	1.22	0.31	1.3	1.52	3.21	6.17	0.45	0
E19	0.18	0.95	0.75	0.88	0.85	0.1	0.87	0.67	0.39	1.11	0.71	1.16	1.31	2.65	5.5	0.52	0.03
E18	0.08	0.84	0.95	0.19	0.78	0.38	0.36	1.11	0.53	1.16	2.4	2.55	0.23	1.36	3.24	1.13	0.86
E17	1.23	0.09	0.51	0.02	0.01	0.01	0.08	1.64	0.8	1.14	1.45	1.62	0.64	1.76	2.05	0.91	1.01
E16	0.96	0.14	0.75	0.13	0.08	0.85	0.01	5.03	0.98	1.03	8.91	2.22	0.54	1.39	1.97	0.91	1.5
E15	13.39	0	0.01	0.03	0.09	0.3	0	1.96	0.97	1	1.04	0.71	0.45	0.6	0.6	1.15	1.38
E14	2.3	0.99	0.23	0.93	0.02	0.85	0	3.3	0.22	1.14	1.85	1.28	0.39	1.79	0.98	0.91	1.46
E13	0.52	0.82	0.72	0.88	0.84	0.85	0.01	1.32	2.97	0.94	0.93	0.95	0.36	0.63	0.62	1.06	1.7
E12	5.63	0	0.11	0.13	0.74	0.01	0	1.78	0.41	0.84	0.34	0.48	0.24	0.2	0.44	1.07	2.29
E11	3.12	0.2	0.8	0.91	0.99	0.02	0	1.22	0.88	0.94	0.47	0.6	0.27	0.3	0.56	1.18	2.29
E10	2.4	0.1	0.34	0.61	0.31	0.03	0	2.39	0.8	1.12	0.68	0.58	0.36	0.58	0.78	1.33	1.49
E9	0.99	0.79	0.14	0.57	0.8	0.11	0	0.88	0.4	1.08	0.56	0.69	0.32	0.71	0.56	1.23	1.46
E8	56.3	0	0	0	0	0	0	0.32	1.28	0.96	0.74	0.93	1.17	0.75	0.5	1.04	0.79
E7	2.05	0.93	0.06	0	0.01	0.06	0	1.95	0.36	1.14	3.13	1.54	0.62	1.97	1.26	0.84	1.05
E6	0.53	0.89	0.11	0	0.01	0.05	0.96	1.98	0.09	1.22	0.97	2.3	1.86	3.72	5.64	0.23	0.01
E5	0.82	0.01	0.05	0.17	0.31	0.47	0.79	3.39	0.59	1.14	0.72	1.19	2.4	2.68	5.15	0.56	0.12
E4	5.31	0	0.01	0	0	0.01	0.94	0.64	0.13	1.2	0.31	1.77	2.5	3.53	4.66	0.29	0.01
E3	0.51	1	0.99	0.02	0	0.91	0.96	4.83	0.18	1.25	6.22	4.21	1.12	3.52	5.89	0.36	0.06
E2	1.92	1	0.77	0.95	0.09	0.05	0.33	0.89	0.44	1.19	1.22	1.15	0.75	1.69	1.83	1.02	1.03
E1	0.84	0.95	0.99	0.14	0.02	0.12	0.02	2.75	0.25	1.21	9.46	3.35	0.92	2.48	3.24	0.74	0.7

Figure S5. Features of chromatin states and genome occupancy in brain of the workers of *A.m.mellifera*. Columns from left to right - Occupied genome fraction (purple), Features comprising states (in blue), and relative enrichment of respective genomic regions (in green).

Table S1. ChromHMM chromatin states interpretation.

**E1**	Inactive TSS	**E13**	Repeat-enriched
**E2**	Introns (low expression)	**E14**	Active enhancers
**E3**	Active TSS	**E15**	Inactive, but accessible chromatin
**E4**	Transcription noise	**E16**	Repressed TSS
**E5**	Transcription noise/transition state	**E17**	Region enriched with H3K4me3
**E6**	Transcribed genes	**E18**	Deacitvating promoter (transition)
**E7**	Inactive CRE	**E19**	Poised promoter
**E8**	Silent domains/heterochromatin	**E20**	High-expressed genes
**E9**	Poised enhancers	**E21**	Active enhancers
**E10**	Transition state	**E22**	Introns (high expresion)
**E11**	PC-repressed CRE	**E23**	Deactivating TSS (transition)
**E12**	PC-repressed genes		

Table S2. Repeats, which serve as cluster markers in sc-RNA-seq analysis. Average expression shows the mean counts by all cells in a given cluster. Percent expressed shows the portion of cells expressing the repeat.

Average Expression	Percent Expressed	REs	Cluster	Average Expression scaled
1,25	11,85	Unknown-1/2	6	1,5
1,16	15,51	Unknown-1/2	7	1,25
1,26	24,92	Unknown-1/2	13	1,54
0,51	26,15	Unknown-4/303	11	1,94
0,5	24,51	Unknown-4/303	12	1,89
0,54	33,17	Unknown-4/303	16	2,27
0,98	9,26	Unknown-5/2258	3	1,18
1,21	22,79	Unknown-5/2258	8	1,95
1,03	20,43	Unknown-5/2258	9	1,33
0,78	33,51	Copia-5/1071	11	2,5
0,61	9,32	Copia-5/1071	17	1,43
2,54	22,54	Unknown-6/719	4	1,3
2,61	19,81	Unknown-6/719	10	1,41
3,5	35,18	Unknown-6/719	17	2,5
8,51	48,26	EnSpm-5/1919	10	2,02
10,41	66,64	EnSpm-5/1919	17	2,5
7,8	58,94	Unknown-1/33	10	1,31
13,02	81,33	Unknown-1/33	17	2,5
2,53	44,94	Mariner-1/1	7	2,45
1,92	52,49	Mariner-1/1	13	1,06
